# Medical Opinion and Movement

**Published:** 1912-03-30

**Authors:** 


### MEDICAL OPINION AND MOVEMENT.

### A New Treatment for Chorea.

Dr. Rocaz has reported to the Bordeaux Medical and Surgical Society the history of four cases of grave chorea rapidly cured by subarachnoid injections of magnesium sulphate. His method was as follows: After having drawn off 10 c.c. of cerebro-spinal fluid, he injected 2 c.c. of a 25 per cent. solution of magnesium sulphate. This procedure has rapid results, the choreic movements disappearing in a few days. It has, however, certain disadvantages, consisting of more or less violent headache, lumbar pains, and pains in the lower limbs. A preliminary injection of atropine will however prevent, or at any rate reduce, these disadvantages. More alarming, though equally harmless in the end, is the occasional appearance of hemiparesis or of torpor. The author has never noticed any renal complications as the result of this treatment, although in one of his cases the child had previously had an attack of nephritis after a dose of antipyrin.

### The Electro-magnet in General Surgery.

At a meeting of the Société de Médecine du Departement du Nord, Dr. Bettremieux drew attention to the uses of the electro-magnet in general surgery. The instrument has a well-recognised place in the extraction of foreign bodies from the eye, but seems to have been overlooked when dealing with cases of foreign bodies in other parts of the human frame. It cannot be denied, however, that in many cases where pieces of needle have penetrated the tissues of the limbs, extraction is frequently difficult even when the position of the fragment has been accurately located by $x$-rays. In many cases a general anæsthetic is necessary before the foreign body can be removed. As the result of the author's experience with the electro-magnet, he comes to the conclusion that extensive incisions and general anæsthetics often can be avoided. The limb should be subjected to the $x$-rays and the needle located. The magnet should then be applied over the spot where the fragment would be looked for. Sometimes more than one application may be necessary, as the first one may only alter the position of the needle. When the fragment is fairly superficial the skin over the point where the needle would tend to emerge will be seen to rise up in front of the fragment. A drop of tincture of iodine is applied to this point and then a small incision is made to allow of the exit of the needle. The wound can then be dressed in the ordinary way. In some cases where the fragment is deeply imbedded it may be necessary to make a more lengthy incision, so as to allow of the magnet coming nearer the foreign body, but even in such cases considerable help can be derived from the magnet, and as no lengthy manipulations are required, the operation can be carried out under local anæsthesia.

### The Transmission of Leprosy.

One after another many diseases whose methods of transmission were mysterious have been shown to be carried from one patient to the next by insect activity. The mosquito for malaria and yellow fever, the tsetse for sleeping-sickness, are household words, and the tick and the flea are equally potent in transmitting spirilla and bacillus pestis. The sandfly is suspected by Dr. Sambon of being the vehicle of pellagra, and Dr. Lindsay Sandes, Research Officer at Robben Island, has suggested that the bed-bug may quite possibly convey the contagion of leprosy. This author contributed to the *Journal of Tropical Medicine and Hygiene* the experiments which lead him to this conclusion. He fed respectively seventy flies, eighty mosquitoes, sixty fleas, and seventy-five bugs on ulcerated leprotic surfaces, chiefly of the forearm; all the insects had been previously starved. Half an hour later smears were made from the alimentary canal, and acid-fast bacilli searched for. Only three bacilli were thus found among the fleas, three among the flies, and three among the mosquitoes, distributed amongst five insects. But twenty of the bugs contained such bacilli in abundance; normally there are no acid-fast bacilli in the bug. These bacilli can be found up to twenty days from their imbibition. One bug which died was found to be swarming with such bacilli in all the tissues; the author believes the bug died of acute leprosy. Experiments on animals have hitherto failed of any definite result, but the clue is one well worth following up. Leprosy is almost solely a disease of uncleanly people, and it may be the progress of hygiene that is abolishing it in Europe as much as segregation laws.

